# [1,5-Bis(4-fluoro­phen­yl)thio­carbazo­nato-κ^2^ *N* ^5^,*S*]phenyl­mercury(II) dichloro­methane hemisolvate

**DOI:** 10.1107/S1600536811050331

**Published:** 2011-11-30

**Authors:** Karel G. von Eschwege, Fabian Muller, Alfred Muller

**Affiliations:** a Department of Chemistry, University of the Free State, PO Box 339, Bloemfontein 9300, South Africa; b Research Center for Synthesis and Catalysis, Department of Chemistry, University of Johannesburg (APK Campus), PO Box 524, Auckland Park, Johannesburg 2006, South Africa

## Abstract

In the title compound, [Hg(C_6_H_5_)(C_13_H_9_F_2_N_4_S)]·0.5CH_2_Cl_2_, the Hg(C_6_H_5_) units are twisted out of the planes of the thio­carbazo­nate ligands by 61.49 (10) and 67.79 (11)° in the two complex mol­ecules comprising the asymmetric unit. Important geometrical parameters include Hg—C = 2.079 (4) and 2.087 (4) Å, Hg—S = 2.3869 (10) and 2.3889 (11) Å, and C—Hg—S = 166.42 (12) and 168.09 (13)°. Weak intramolecular Hg—N bonding inter­actions of 2.589 (4) and 2.626 (4) Å are observed. In the crystal, C—H⋯Cl, C—H⋯F, C—H⋯N, C—H⋯π and π–π [centroid–centroid distances = 3.648 (3) and 3.641 (3) Å] inter­actions, create parallel planes along [101].

## Related literature

For general background to thio­carbodiazo­natomercury(II) complexes, see: Irving *et al.* (1949 ▶); Webb *et al.* (1950 ▶); von Eschwege *et al.* (2011 ▶). For synthetic procedures relating to the title compound, see: Mirkhalaf *et al.* (1998 ▶); von Eschwege *et al.* (2008 ▶). For details of the superimposed fitting of structures with *Mercury*, see: Weng *et al.* (2008*a* ▶,*b* ▶).
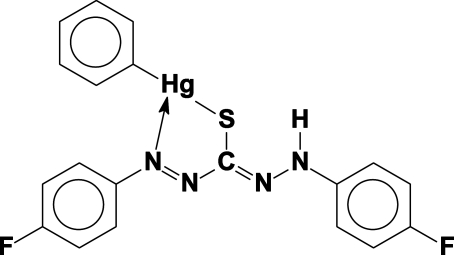


## Experimental

### 

#### Crystal data

[Hg(C_6_H_5_)(C_13_H_9_F_2_N_4_S)]·0.5CH_2_Cl_2_
*M* *_r_* = 611.46
Monoclinic, 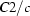

*a* = 31.996 (3) Å
*b* = 10.1889 (9) Å
*c* = 26.892 (2) Å
β = 116.818 (1)°
*V* = 7823.8 (12) Å^3^
*Z* = 16
Mo *K*α radiation
μ = 8.14 mm^−1^
*T* = 100 K
0.5 × 0.41 × 0.12 mm

#### Data collection

Bruker APEX DUO 4K CCD diffractometer
Absorption correction: multi-scan (*SADABS*; Bruker, 2008 ▶) *T*_min_ = 0.106, *T*_max_ = 0.441
94461 measured reflections
9726 independent reflections
8965 reflections with *I* > 2σ(*I*)
*R*_int_ = 0.045

#### Refinement

*R*[*F* ^2^ > 2σ(*F* ^2^)] = 0.032
*wR*(*F* ^2^) = 0.074
*S* = 1.21
9726 reflections
514 parameters
H-atom parameters constrained
Δρ_max_ = 3.10 e Å^−3^
Δρ_min_ = −2.47 e Å^−3^

### 

Data collection: *APEX2* (Bruker, 2011 ▶); cell refinement: *SAINT* (Bruker, 2008 ▶); data reduction: *SAINT* and *XPREP* (Bruker, 2008 ▶); program(s) used to solve structure: *SIR97* (Altomare *et al.*, 1999 ▶); program(s) used to refine structure: *SHELXL97* (Sheldrick, 2008 ▶); molecular graphics: *DIAMOND* (Brandenburg & Putz, 2005 ▶); software used to prepare material for publication: *WinGX* (Farrugia, 1999 ▶).

## Supplementary Material

Crystal structure: contains datablock(s) global, I. DOI: 10.1107/S1600536811050331/zq2140sup1.cif

Structure factors: contains datablock(s) I. DOI: 10.1107/S1600536811050331/zq2140Isup2.hkl

Additional supplementary materials:  crystallographic information; 3D view; checkCIF report

## Figures and Tables

**Table 1:** Hydrogen-bond geometry (Å, °) *Cg*1 and *Cg*2 are the centroids of the C2–C7 and C8–C13 rings, respectively.

*D*—H⋯*A*	*D*—H	H⋯*A*	*D*⋯*A*	*D*—H⋯*A*
C28—H28⋯Cl1	0.95	2.77	3.598 (5)	146
C31—H31⋯F4^i^	0.95	2.53	3.413 (6)	155
C39—H39*A*⋯N2	0.99	2.62	3.558 (6)	158
C7—H7⋯*Cg*1^ii^	0.95	2.54	3.451 (5)	162
C12—H12⋯*Cg*2^iii^	0.95	2.70	3.516 (6)	144
C26—H26⋯*Cg*2^ii^	0.95	2.69	3.500 (5)	144

Symmetry codes: (i) 
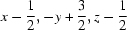
; (ii) 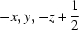; (iii) 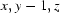.

**Fig. 1.**
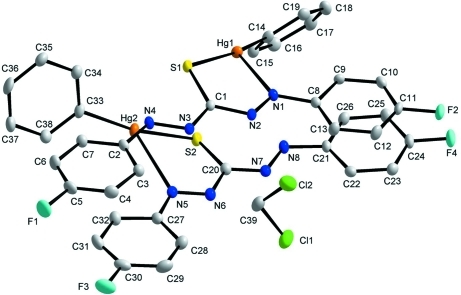
View of the title compound indicating labelling and displacement ellipsoids (drawn at a 50% probability level).

**Fig. 2.**
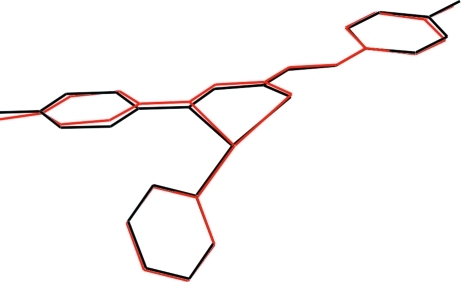
Superimposed drawing of the two crystallographically independent mercury(II) molecules of the crystal structure.

**Fig. 3.**
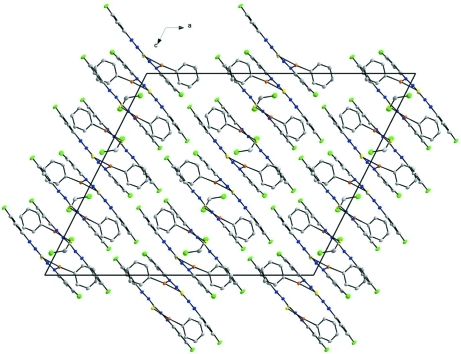
Packing diagram of the title compound showing the parallel packing arrangement along the [101] direction.

## supplementary crystallographic information

### Comment

With the aim of investigating the influence of electron withdrawing groups on the photochromic (Irving *et al.*, 1949; Webb *et al.*, 1950) and redox reactions (von Eschwege *et al.*, 2011) of dithizonatophenylmercury(II) complexes, a series of halogenated dithizones were synthesized and for the first time complexed with mercury. Deep orange-red needle crystals of the *para*-fluoro derivative, suitable for X-ray crystallography, were isolated from a dichloromethane solution overlaid with ethanol.

The asymmetric unit of the title compound contains two crystallographically independent mercury(II) molecules and one solvent molecule of dichloromethane (Fig. 1). Geometrical parameters of the two dithizonato complexes are fairly similar with Hg—C = 2.079 (4) / 2.087 (4) Å; Hg—S = 2.3869 (10) / 2.3889 (11) Å; and C—Hg—S = 166.42 (12)/ 168.09 (13)° for Hg1 and Hg2, respectively. The mercury coordination environments differ slightly and can be seen most prominently from the dihedral angles between the metal coordination plane *vs.* the plane formed by the dithizonato ligands (19.03 (8)° *vs.* 23.45 (8)° for Hg1 and Hg2, respectively). Differences between the two units are illustrated in Fig. 2 with a superimposed fit using Mercury (Weng *et al.*, 2008*a*; Weng *et al.*, 2008*b*). The root mean square deviation (RMSD) was calculated as 0.151 Å, and the maximum distance between two atoms = 0.333 Å.

Several interactions C—H···*X* (*X* = Cl, F, N), C—H···*Cg* (Table 1) and *Cg*···*Cg* (Table 2) stabilizes the crystal packing, creating parallel planes along the [101] direction (Fig. 3).

### Experimental

Solvents (AR) purchased from Merck and reagents from Sigma-Aldrich were used without further purification. The *para*-fluoro derivative of dithizone, (*p*-FPhNHN)_2_CS), was prepared according to the procedure reported by Mirkhalaf *et al.* (1998). The synthesis and crystallization of the title compound was done according to a procedure earlier reported by von Eschwege *et al.* (2008).

### Refinement

All hydrogen atoms were positioned in geometrically idealized positions with C—H = 0.95 Å (aromatic) or 0.88 Å (methylene) and N—H = 0.86 Å (imine). All hydrogen atoms were allowed to ride on their parent atoms with *U*_iso_(H) = 1.2*U*_eq_(C/N). The highest residual electron density of 3.10 e.Å^-3^ is 1.13 Å from Hg2 and the deepest hole of -2.47 e.Å^-3^ is 0.89 Å from Hg1. Both represent no physical meaning. Several discrepant reflections were omitted (see _iucr_refine_instructions_details).

### Crystal data

**Table:**

[Hg(C_6_H_5_)(C_13_H_9_F_2_N_4_S)]·0.5CH_2_Cl_2_	*F*(000) = 4656
*M**_r_* = 611.46	*D*_x_ = 2.076 Mg m^−^^3^
Monoclinic, *C*2/*c*	Mo *K*α radiation, λ = 0.71073 Å
Hall symbol: -C 2yc	Cell parameters from 9279 reflections
*a* = 31.996 (3) Å	θ = 2.7–28.3°
*b* = 10.1889 (9) Å	µ = 8.14 mm^−^^1^
*c* = 26.892 (2) Å	*T* = 100 K
β = 116.818 (1)°	Plate, red
*V* = 7823.8 (12) Å^3^	0.5 × 0.41 × 0.12 mm
*Z* = 16	

### Data collection

**Table:**

Bruker APEX DUO 4K CCD diffractometer	9726 independent reflections
graphite	8965 reflections with *I* > 2σ(*I*)
Detector resolution: 8.4 pixels mm^-1^	*R*_int_ = 0.045
φ and ω scans	θ_max_ = 28.3°, θ_min_ = 1.4°
Absorption correction: multi-scan (*SADABS*; Bruker, 2008)	*h* = −42→42
*T*_min_ = 0.106, *T*_max_ = 0.441	*k* = −13→13
94461 measured reflections	*l* = −35→35

### Refinement

**Table:**

Refinement on *F*^2^	Primary atom site location: structure-invariant direct methods
Least-squares matrix: full	Secondary atom site location: difference Fourier map
*R*[*F*^2^ > 2σ(*F*^2^)] = 0.032	Hydrogen site location: inferred from neighbouring sites
*wR*(*F*^2^) = 0.074	H-atom parameters constrained
*S* = 1.21	*w* = 1/[σ^2^(*F*_o_^2^) + (0.0197*P*)^2^ + 88.7759*P*] where *P* = (*F*_o_^2^ + 2*F*_c_^2^)/3
9726 reflections	(Δ/σ)_max_ = 0.003
514 parameters	Δρ_max_ = 3.10 e Å^−^^3^
0 restraints	Δρ_min_ = −2.47 e Å^−^^3^

### Special details

**Table:**

Experimental. The intensity data was collected on a Bruker Apex DUO 4 K CCD diffractometer using an exposure time of 10 s/frame. A total of 2980 frames were collected with a frame width of 0.5° covering up to θ = 28.33° with 99.5% completeness accomplished.Analytical data: *M*.p. 208 °C; λ_max_ (dichloromethane) 471 nm; ^1^H (300 MHz, CDCl_3_) 7.06 – 7.99 (13 H, m, 2 × C_6_H_4_F & 1 × C_6_H_5_).
Geometry. All e.s.d.'s (except the e.s.d. in the dihedral angle between two l.s. planes) are estimated using the full covariance matrix. The cell e.s.d.'s are taken into account individually in the estimation of e.s.d.'s in distances, angles and torsion angles; correlations between e.s.d.'s in cell parameters are only used when they are defined by crystal symmetry. An approximate (isotropic) treatment of cell e.s.d.'s is used for estimating e.s.d.'s involving l.s. planes.
Refinement. Refinement of *F*^2^ against ALL reflections. The weighted *R*-factor *wR* and goodness of fit *S* are based on *F*^2^, conventional *R*-factors *R* are based on *F*, with *F* set to zero for negative *F*^2^. The threshold expression of *F*^2^ > σ(*F*^2^) is used only for calculating *R*-factors(gt) *etc*. and is not relevant to the choice of reflections for refinement. *R*-factors based on *F*^2^ are statistically about twice as large as those based on *F*, and *R*- factors based on ALL data will be even larger.

### Fractional atomic coordinates and isotropic or equivalent isotropic displacement parameters (Å^2^)

**Table:**

	*x*	*y*	*z*	*U*_iso_*/*U*_eq_	
C1	0.46620 (14)	0.7851 (4)	0.15534 (16)	0.0126 (8)	
C2	0.35329 (14)	0.7322 (4)	0.04266 (17)	0.0150 (8)	
C3	0.34667 (15)	0.5979 (4)	0.03934 (18)	0.0180 (9)	
H3	0.3697	0.5413	0.0655	0.022*	
C4	0.30560 (16)	0.5467 (5)	−0.00303 (19)	0.0215 (9)	
H4	0.3003	0.4547	−0.006	0.026*	
C5	0.27293 (16)	0.6309 (5)	−0.04042 (19)	0.0226 (10)	
C6	0.27872 (15)	0.7644 (5)	−0.03753 (19)	0.0214 (9)	
H6	0.2554	0.8203	−0.0637	0.026*	
C7	0.31949 (15)	0.8161 (5)	0.00454 (18)	0.0175 (9)	
H7	0.3244	0.9083	0.0074	0.021*	
C8	0.57739 (14)	0.6773 (4)	0.25709 (17)	0.0148 (8)	
C9	0.61666 (14)	0.7429 (4)	0.29689 (17)	0.0148 (8)	
H9	0.6175	0.8361	0.2977	0.018*	
C10	0.65435 (15)	0.6717 (5)	0.33513 (17)	0.0183 (9)	
H10	0.6811	0.7149	0.3625	0.022*	
C11	0.65210 (15)	0.5387 (4)	0.33253 (18)	0.0183 (9)	
C12	0.61417 (17)	0.4698 (5)	0.2933 (2)	0.0226 (10)	
H12	0.6141	0.3766	0.2924	0.027*	
C13	0.57615 (16)	0.5418 (4)	0.25539 (19)	0.0195 (9)	
H13	0.5494	0.4977	0.2283	0.023*	
C14	0.60941 (14)	1.1066 (4)	0.25128 (18)	0.0156 (8)	
C15	0.62304 (15)	1.1764 (4)	0.21641 (19)	0.0181 (9)	
H15	0.6017	1.1858	0.1781	0.022*	
C16	0.66723 (16)	1.2325 (5)	0.2365 (2)	0.0200 (9)	
H16	0.6757	1.2804	0.2122	0.024*	
C17	0.69890 (16)	1.2186 (5)	0.2922 (2)	0.0219 (9)	
H17	0.7294	1.255	0.306	0.026*	
C18	0.68571 (16)	1.1510 (5)	0.32781 (19)	0.0211 (9)	
H18	0.7073	1.1417	0.3661	0.025*	
C19	0.64101 (16)	1.0969 (4)	0.30767 (18)	0.0180 (9)	
H19	0.632	1.053	0.3325	0.022*	
N1	0.54016 (12)	0.7614 (4)	0.22209 (14)	0.0145 (7)	
N2	0.50339 (12)	0.7028 (3)	0.18737 (14)	0.0133 (7)	
N3	0.43071 (12)	0.7197 (4)	0.11738 (14)	0.0142 (7)	
N4	0.39362 (12)	0.7908 (4)	0.08385 (14)	0.0157 (7)	
H4A	0.3945	0.8767	0.0877	0.019*	
S1	0.46396 (3)	0.95572 (10)	0.16432 (4)	0.01423 (19)	
Hg1	0.544460 (5)	1.014330 (16)	0.216419 (7)	0.01517 (5)	
C20	0.54721 (15)	0.8218 (5)	0.09362 (18)	0.0172 (8)	
C21	0.65986 (14)	0.7796 (4)	0.20752 (17)	0.0151 (8)	
C22	0.66609 (15)	0.6447 (4)	0.21190 (18)	0.0173 (8)	
H22	0.643	0.5882	0.1858	0.021*	
C23	0.70632 (16)	0.5930 (5)	0.25465 (18)	0.0193 (9)	
H23	0.7113	0.5009	0.258	0.023*	
C24	0.73905 (15)	0.6773 (5)	0.29230 (18)	0.0193 (9)	
C25	0.73349 (15)	0.8114 (5)	0.28909 (19)	0.0190 (9)	
H25	0.7564	0.8671	0.316	0.023*	
C26	0.69359 (15)	0.8636 (5)	0.24555 (19)	0.0185 (9)	
H26	0.6893	0.9559	0.2417	0.022*	
C27	0.43996 (15)	0.6906 (5)	−0.00737 (18)	0.0177 (9)	
C28	0.44574 (16)	0.5554 (5)	−0.0017 (2)	0.0228 (10)	
H28	0.4746	0.5199	0.0251	0.027*	
C29	0.40969 (19)	0.4717 (5)	−0.0349 (2)	0.0281 (11)	
H29	0.4132	0.3791	−0.0312	0.034*	
C30	0.36822 (17)	0.5281 (5)	−0.0739 (2)	0.0240 (10)	
C31	0.36194 (16)	0.6599 (5)	−0.08133 (19)	0.0242 (10)	
H31	0.3334	0.6946	−0.1093	0.029*	
C32	0.39781 (15)	0.7428 (5)	−0.04747 (18)	0.0202 (9)	
H32	0.3938	0.8352	−0.0515	0.024*	
C33	0.39213 (15)	1.1012 (5)	0.01027 (19)	0.0187 (9)	
C34	0.37593 (16)	1.1470 (4)	0.0471 (2)	0.0205 (9)	
H34	0.397	1.1533	0.0856	0.025*	
C35	0.32924 (17)	1.1840 (5)	0.0282 (2)	0.0240 (10)	
H35	0.3186	1.2153	0.0539	0.029*	
C36	0.29825 (16)	1.1749 (5)	−0.0282 (2)	0.0267 (11)	
H36	0.2663	1.198	−0.0411	0.032*	
C37	0.31419 (17)	1.1320 (5)	−0.0653 (2)	0.0257 (10)	
H37	0.2932	1.1272	−0.1039	0.031*	
C38	0.36084 (16)	1.0957 (5)	−0.04644 (19)	0.0211 (9)	
H38	0.3715	1.0671	−0.0724	0.025*	
N5	0.47415 (12)	0.7835 (4)	0.02619 (15)	0.0159 (7)	
N6	0.51276 (13)	0.7329 (4)	0.06020 (15)	0.0183 (8)	
N7	0.58460 (13)	0.7619 (4)	0.13032 (15)	0.0171 (7)	
N8	0.61938 (13)	0.8373 (4)	0.16571 (15)	0.0178 (7)	
H8	0.617	0.9233	0.1629	0.021*	
F1	0.23290 (10)	0.5798 (3)	−0.08167 (13)	0.0331 (7)	
F2	0.68937 (10)	0.4681 (3)	0.36991 (12)	0.0283 (7)	
F3	0.33195 (11)	0.4477 (3)	−0.10507 (13)	0.0363 (8)	
F4	0.77784 (10)	0.6248 (3)	0.33508 (12)	0.0272 (6)	
S2	0.54317 (4)	0.99363 (11)	0.08664 (4)	0.0172 (2)	
Hg2	0.460639 (5)	1.031777 (17)	0.040790 (7)	0.01692 (5)	
C39	0.47037 (19)	0.3912 (5)	0.1214 (2)	0.0325 (12)	
H39A	0.4732	0.4864	0.1295	0.039*	
H39B	0.4408	0.3765	0.0871	0.039*	
Cl1	0.51796 (5)	0.33931 (16)	0.10993 (6)	0.0417 (3)	
Cl2	0.46844 (5)	0.30584 (15)	0.17705 (6)	0.0385 (3)	

### Atomic displacement parameters (Å^2^)

**Table:**

	*U*^11^	*U*^22^	*U*^33^	*U*^12^	*U*^13^	*U*^23^
C1	0.0108 (17)	0.0143 (19)	0.0125 (18)	0.0015 (15)	0.0050 (15)	0.0012 (14)
C2	0.0100 (18)	0.021 (2)	0.0123 (18)	−0.0017 (15)	0.0031 (15)	−0.0010 (15)
C3	0.017 (2)	0.018 (2)	0.0162 (19)	0.0010 (16)	0.0046 (17)	0.0004 (16)
C4	0.021 (2)	0.018 (2)	0.024 (2)	−0.0047 (17)	0.0082 (19)	−0.0049 (17)
C5	0.014 (2)	0.030 (3)	0.017 (2)	−0.0043 (18)	0.0019 (17)	−0.0067 (18)
C6	0.0124 (19)	0.024 (2)	0.020 (2)	−0.0002 (17)	0.0006 (17)	−0.0003 (18)
C7	0.0141 (19)	0.018 (2)	0.017 (2)	−0.0009 (16)	0.0041 (17)	−0.0006 (16)
C8	0.0110 (18)	0.018 (2)	0.0136 (18)	0.0004 (15)	0.0040 (16)	−0.0014 (15)
C9	0.0150 (19)	0.0117 (19)	0.0150 (18)	−0.0004 (15)	0.0042 (16)	−0.0032 (15)
C10	0.0124 (19)	0.025 (2)	0.0130 (18)	0.0003 (17)	0.0021 (16)	−0.0022 (16)
C11	0.0152 (19)	0.019 (2)	0.0175 (19)	0.0085 (16)	0.0046 (17)	0.0034 (16)
C12	0.021 (2)	0.013 (2)	0.028 (2)	0.0040 (17)	0.0063 (19)	0.0022 (17)
C13	0.015 (2)	0.015 (2)	0.023 (2)	0.0010 (16)	0.0035 (17)	−0.0018 (17)
C14	0.0098 (17)	0.0118 (19)	0.023 (2)	−0.0026 (14)	0.0049 (16)	−0.0031 (16)
C15	0.016 (2)	0.013 (2)	0.021 (2)	−0.0006 (16)	0.0047 (17)	−0.0005 (16)
C16	0.018 (2)	0.016 (2)	0.025 (2)	−0.0034 (17)	0.0100 (18)	0.0008 (17)
C17	0.015 (2)	0.017 (2)	0.029 (2)	−0.0030 (16)	0.0056 (19)	−0.0023 (18)
C18	0.019 (2)	0.017 (2)	0.020 (2)	−0.0034 (17)	0.0013 (18)	−0.0019 (17)
C19	0.020 (2)	0.011 (2)	0.020 (2)	−0.0009 (16)	0.0073 (18)	−0.0008 (16)
N1	0.0108 (16)	0.0164 (18)	0.0133 (15)	−0.0001 (13)	0.0027 (13)	0.0005 (13)
N2	0.0096 (15)	0.0137 (17)	0.0142 (16)	0.0000 (13)	0.0032 (13)	0.0009 (13)
N3	0.0118 (16)	0.0141 (17)	0.0140 (16)	0.0005 (13)	0.0035 (14)	0.0007 (13)
N4	0.0112 (16)	0.0156 (18)	0.0145 (16)	−0.0008 (13)	0.0006 (14)	0.0002 (13)
S1	0.0113 (4)	0.0120 (5)	0.0158 (4)	0.0002 (3)	0.0030 (4)	0.0009 (4)
Hg1	0.01147 (7)	0.01376 (8)	0.01828 (8)	−0.00258 (5)	0.00496 (6)	−0.00227 (6)
C20	0.0146 (19)	0.019 (2)	0.0162 (19)	0.0007 (16)	0.0054 (17)	0.0003 (16)
C21	0.0112 (18)	0.019 (2)	0.0137 (18)	0.0025 (15)	0.0041 (16)	0.0023 (15)
C22	0.0142 (19)	0.020 (2)	0.0155 (19)	0.0001 (16)	0.0052 (16)	−0.0032 (16)
C23	0.020 (2)	0.016 (2)	0.020 (2)	0.0028 (17)	0.0070 (18)	0.0005 (16)
C24	0.0128 (19)	0.024 (2)	0.0163 (19)	0.0040 (17)	0.0025 (17)	0.0042 (17)
C25	0.0123 (19)	0.021 (2)	0.020 (2)	−0.0021 (16)	0.0038 (17)	0.0001 (17)
C26	0.0148 (19)	0.016 (2)	0.022 (2)	0.0014 (16)	0.0065 (17)	0.0006 (17)
C27	0.0136 (19)	0.022 (2)	0.0164 (19)	0.0006 (16)	0.0056 (17)	−0.0018 (16)
C28	0.017 (2)	0.022 (2)	0.026 (2)	0.0026 (18)	0.0071 (19)	−0.0016 (18)
C29	0.029 (3)	0.020 (2)	0.037 (3)	−0.002 (2)	0.016 (2)	−0.009 (2)
C30	0.023 (2)	0.030 (3)	0.021 (2)	−0.009 (2)	0.0118 (19)	−0.0111 (19)
C31	0.016 (2)	0.037 (3)	0.015 (2)	−0.0026 (19)	0.0024 (17)	0.0001 (19)
C32	0.015 (2)	0.023 (2)	0.019 (2)	0.0002 (17)	0.0049 (17)	0.0016 (17)
C33	0.0117 (18)	0.019 (2)	0.023 (2)	0.0028 (16)	0.0065 (17)	0.0044 (17)
C34	0.018 (2)	0.016 (2)	0.022 (2)	0.0022 (16)	0.0047 (18)	0.0013 (17)
C35	0.021 (2)	0.020 (2)	0.029 (2)	0.0062 (18)	0.010 (2)	0.0000 (19)
C36	0.014 (2)	0.026 (3)	0.034 (3)	0.0089 (18)	0.006 (2)	0.004 (2)
C37	0.019 (2)	0.026 (3)	0.022 (2)	0.0067 (19)	0.0014 (19)	0.0052 (19)
C38	0.020 (2)	0.023 (2)	0.018 (2)	0.0038 (18)	0.0073 (18)	0.0030 (17)
N5	0.0128 (16)	0.0198 (19)	0.0147 (16)	0.0020 (14)	0.0060 (14)	0.0011 (14)
N6	0.0127 (16)	0.022 (2)	0.0168 (17)	0.0016 (14)	0.0037 (14)	−0.0004 (14)
N7	0.0133 (16)	0.0204 (19)	0.0154 (17)	0.0021 (14)	0.0046 (14)	−0.0011 (14)
N8	0.0141 (17)	0.0177 (18)	0.0163 (17)	0.0013 (14)	0.0022 (14)	0.0011 (14)
F1	0.0203 (14)	0.0312 (17)	0.0299 (15)	−0.0072 (12)	−0.0044 (12)	−0.0097 (13)
F2	0.0210 (14)	0.0263 (15)	0.0261 (14)	0.0126 (12)	0.0005 (12)	0.0025 (12)
F3	0.0330 (17)	0.0390 (19)	0.0339 (17)	−0.0150 (14)	0.0124 (14)	−0.0188 (14)
F4	0.0171 (13)	0.0257 (15)	0.0253 (14)	0.0048 (11)	−0.0023 (11)	0.0051 (12)
S2	0.0127 (5)	0.0169 (5)	0.0192 (5)	0.0007 (4)	0.0048 (4)	0.0027 (4)
Hg2	0.01199 (8)	0.01754 (9)	0.01911 (8)	0.00276 (6)	0.00514 (6)	0.00486 (6)
C39	0.026 (3)	0.025 (3)	0.034 (3)	0.009 (2)	0.004 (2)	−0.006 (2)
Cl1	0.0363 (7)	0.0410 (8)	0.0430 (8)	0.0185 (6)	0.0138 (6)	0.0053 (6)
Cl2	0.0435 (8)	0.0335 (7)	0.0314 (6)	0.0023 (6)	0.0107 (6)	−0.0066 (5)

### Geometric parameters (Å, °)

**Table:**

C1—N3	1.315 (5)	C20—S2	1.759 (5)
C1—N2	1.391 (5)	C21—C22	1.386 (6)
C1—S1	1.761 (4)	C21—C26	1.395 (6)
C2—C3	1.382 (6)	C21—N8	1.405 (5)
C2—C7	1.397 (6)	C22—C23	1.386 (6)
C2—N4	1.400 (5)	C22—H22	0.95
C3—C4	1.395 (6)	C23—C24	1.379 (6)
C3—H3	0.95	C23—H23	0.95
C4—C5	1.375 (7)	C24—F4	1.364 (5)
C4—H4	0.95	C24—C25	1.376 (7)
C5—F1	1.364 (5)	C25—C26	1.391 (6)
C5—C6	1.370 (7)	C25—H25	0.95
C6—C7	1.388 (6)	C26—H26	0.95
C6—H6	0.95	C27—C28	1.389 (7)
C7—H7	0.95	C27—C32	1.397 (6)
C8—C13	1.382 (6)	C27—N5	1.420 (6)
C8—C9	1.399 (5)	C28—C29	1.387 (7)
C8—N1	1.423 (5)	C28—H28	0.95
C9—C10	1.385 (6)	C29—C30	1.391 (7)
C9—H9	0.95	C29—H29	0.95
C10—C11	1.357 (7)	C30—F3	1.357 (5)
C10—H10	0.95	C30—C31	1.360 (8)
C11—F2	1.367 (5)	C31—C32	1.384 (6)
C11—C12	1.387 (6)	C31—H31	0.95
C12—C13	1.392 (6)	C32—H32	0.95
C12—H12	0.95	C33—C34	1.389 (7)
C13—H13	0.95	C33—C38	1.398 (6)
C14—C15	1.394 (6)	C33—Hg2	2.087 (4)
C14—C19	1.396 (6)	C34—C35	1.396 (6)
C14—Hg1	2.079 (4)	C34—H34	0.95
C15—C16	1.388 (6)	C35—C36	1.391 (7)
C15—H15	0.95	C35—H35	0.95
C16—C17	1.386 (6)	C36—C37	1.380 (8)
C16—H16	0.95	C36—H36	0.95
C17—C18	1.390 (7)	C37—C38	1.393 (6)
C17—H17	0.95	C37—H37	0.95
C18—C19	1.394 (6)	C38—H38	0.95
C18—H18	0.95	N5—N6	1.268 (5)
C19—H19	0.95	N5—Hg2	2.626 (4)
N1—N2	1.273 (5)	N7—N8	1.334 (5)
N1—Hg1	2.589 (4)	N8—H8	0.88
N3—N4	1.334 (5)	S2—Hg2	2.3889 (11)
N4—H4A	0.88	C39—Cl2	1.757 (6)
S1—Hg1	2.3869 (10)	C39—Cl1	1.765 (6)
C20—N7	1.309 (5)	C39—H39A	0.99
C20—N6	1.396 (6)	C39—H39B	0.99
			
N3—C1—N2	111.9 (4)	C22—C21—C26	120.8 (4)
N3—C1—S1	122.1 (3)	C22—C21—N8	121.9 (4)
N2—C1—S1	125.9 (3)	C26—C21—N8	117.3 (4)
C3—C2—C7	120.9 (4)	C23—C22—C21	119.4 (4)
C3—C2—N4	122.2 (4)	C23—C22—H22	120.3
C7—C2—N4	116.8 (4)	C21—C22—H22	120.3
C2—C3—C4	118.9 (4)	C24—C23—C22	119.1 (4)
C2—C3—H3	120.5	C24—C23—H23	120.5
C4—C3—H3	120.5	C22—C23—H23	120.5
C5—C4—C3	119.3 (4)	F4—C24—C25	119.0 (4)
C5—C4—H4	120.4	F4—C24—C23	118.4 (4)
C3—C4—H4	120.4	C25—C24—C23	122.6 (4)
F1—C5—C6	118.6 (4)	C24—C25—C26	118.4 (4)
F1—C5—C4	118.8 (4)	C24—C25—H25	120.8
C6—C5—C4	122.6 (4)	C26—C25—H25	120.8
C5—C6—C7	118.4 (4)	C25—C26—C21	119.7 (4)
C5—C6—H6	120.8	C25—C26—H26	120.1
C7—C6—H6	120.8	C21—C26—H26	120.1
C6—C7—C2	119.8 (4)	C28—C27—C32	119.8 (4)
C6—C7—H7	120.1	C28—C27—N5	124.4 (4)
C2—C7—H7	120.1	C32—C27—N5	115.8 (4)
C13—C8—C9	120.3 (4)	C29—C28—C27	120.5 (4)
C13—C8—N1	125.2 (4)	C29—C28—H28	119.7
C9—C8—N1	114.4 (4)	C27—C28—H28	119.7
C10—C9—C8	119.9 (4)	C28—C29—C30	117.7 (5)
C10—C9—H9	120.1	C28—C29—H29	121.2
C8—C9—H9	120.1	C30—C29—H29	121.2
C11—C10—C9	118.5 (4)	F3—C30—C31	118.5 (5)
C11—C10—H10	120.8	F3—C30—C29	118.4 (5)
C9—C10—H10	120.8	C31—C30—C29	123.1 (4)
C10—C11—F2	118.6 (4)	C30—C31—C32	118.8 (4)
C10—C11—C12	123.5 (4)	C30—C31—H31	120.6
F2—C11—C12	117.8 (4)	C32—C31—H31	120.6
C11—C12—C13	117.8 (4)	C31—C32—C27	120.0 (5)
C11—C12—H12	121.1	C31—C32—H32	120
C13—C12—H12	121.1	C27—C32—H32	120
C8—C13—C12	120.0 (4)	C34—C33—C38	118.5 (4)
C8—C13—H13	120	C34—C33—Hg2	119.8 (3)
C12—C13—H13	120	C38—C33—Hg2	121.6 (4)
C15—C14—C19	118.3 (4)	C33—C34—C35	121.0 (4)
C15—C14—Hg1	118.7 (3)	C33—C34—H34	119.5
C19—C14—Hg1	123.0 (3)	C35—C34—H34	119.5
C16—C15—C14	121.3 (4)	C36—C35—C34	119.8 (5)
C16—C15—H15	119.3	C36—C35—H35	120.1
C14—C15—H15	119.3	C34—C35—H35	120.1
C17—C16—C15	119.9 (4)	C37—C36—C35	119.7 (4)
C17—C16—H16	120	C37—C36—H36	120.2
C15—C16—H16	120	C35—C36—H36	120.2
C16—C17—C18	119.6 (4)	C36—C37—C38	120.4 (4)
C16—C17—H17	120.2	C36—C37—H37	119.8
C18—C17—H17	120.2	C38—C37—H37	119.8
C17—C18—C19	120.4 (4)	C37—C38—C33	120.6 (5)
C17—C18—H18	119.8	C37—C38—H38	119.7
C19—C18—H18	119.8	C33—C38—H38	119.7
C18—C19—C14	120.5 (4)	N6—N5—C27	114.1 (4)
C18—C19—H19	119.8	N6—N5—Hg2	117.1 (3)
C14—C19—H19	119.8	C27—N5—Hg2	127.3 (3)
N2—N1—C8	115.1 (4)	N5—N6—C20	115.5 (4)
N2—N1—Hg1	118.6 (3)	C20—N7—N8	117.0 (4)
C8—N1—Hg1	125.9 (3)	N7—N8—C21	120.1 (4)
N1—N2—C1	114.9 (4)	N7—N8—H8	119.9
C1—N3—N4	116.3 (4)	C21—N8—H8	119.9
N3—N4—C2	121.6 (4)	C20—S2—Hg2	103.17 (15)
N3—N4—H4A	119.2	C33—Hg2—S2	168.09 (13)
C2—N4—H4A	119.2	C33—Hg2—N5	118.83 (15)
C1—S1—Hg1	103.15 (14)	S2—Hg2—N5	72.67 (8)
C14—Hg1—S1	166.42 (12)	Cl2—C39—Cl1	111.4 (3)
C14—Hg1—N1	119.51 (14)	Cl2—C39—H39A	109.3
S1—Hg1—N1	73.36 (8)	Cl1—C39—H39A	109.3
N7—C20—N6	111.7 (4)	Cl2—C39—H39B	109.3
N7—C20—S2	122.9 (3)	Cl1—C39—H39B	109.3
N6—C20—S2	125.3 (3)	H39A—C39—H39B	108
			
C7—C2—C3—C4	0.3 (7)	C26—C21—C22—C23	0.1 (7)
N4—C2—C3—C4	−179.8 (4)	N8—C21—C22—C23	−178.7 (4)
C2—C3—C4—C5	0.2 (7)	C21—C22—C23—C24	0.7 (7)
C3—C4—C5—F1	180.0 (4)	C22—C23—C24—F4	178.2 (4)
C3—C4—C5—C6	−0.8 (8)	C22—C23—C24—C25	−0.2 (7)
F1—C5—C6—C7	180.0 (4)	F4—C24—C25—C26	−179.4 (4)
C4—C5—C6—C7	0.7 (8)	C23—C24—C25—C26	−1.1 (7)
C5—C6—C7—C2	−0.2 (7)	C24—C25—C26—C21	1.9 (7)
C3—C2—C7—C6	−0.3 (7)	C22—C21—C26—C25	−1.4 (7)
N4—C2—C7—C6	179.7 (4)	N8—C21—C26—C25	177.5 (4)
C13—C8—C9—C10	−0.6 (7)	C32—C27—C28—C29	−1.2 (8)
N1—C8—C9—C10	177.0 (4)	N5—C27—C28—C29	177.4 (5)
C8—C9—C10—C11	0.4 (7)	C27—C28—C29—C30	0.6 (8)
C9—C10—C11—F2	179.5 (4)	C28—C29—C30—F3	−177.2 (5)
C9—C10—C11—C12	0.5 (8)	C28—C29—C30—C31	1.1 (8)
C10—C11—C12—C13	−1.2 (8)	F3—C30—C31—C32	176.2 (4)
F2—C11—C12—C13	179.8 (4)	C29—C30—C31—C32	−2.1 (8)
C9—C8—C13—C12	−0.2 (7)	C30—C31—C32—C27	1.4 (7)
N1—C8—C13—C12	−177.5 (4)	C28—C27—C32—C31	0.1 (7)
C11—C12—C13—C8	1.0 (7)	N5—C27—C32—C31	−178.5 (4)
C19—C14—C15—C16	1.5 (7)	C38—C33—C34—C35	1.5 (7)
Hg1—C14—C15—C16	−175.5 (3)	Hg2—C33—C34—C35	−174.8 (4)
C14—C15—C16—C17	0.6 (7)	C33—C34—C35—C36	0.1 (8)
C15—C16—C17—C18	−1.5 (7)	C34—C35—C36—C37	−1.4 (8)
C16—C17—C18—C19	0.3 (7)	C35—C36—C37—C38	1.1 (8)
C17—C18—C19—C14	1.8 (7)	C36—C37—C38—C33	0.5 (8)
C15—C14—C19—C18	−2.7 (7)	C34—C33—C38—C37	−1.8 (7)
Hg1—C14—C19—C18	174.2 (3)	Hg2—C33—C38—C37	174.4 (4)
C13—C8—N1—N2	0.5 (6)	C28—C27—N5—N6	4.7 (7)
C9—C8—N1—N2	−176.9 (4)	C32—C27—N5—N6	−176.7 (4)
C13—C8—N1—Hg1	−172.6 (4)	C28—C27—N5—Hg2	−160.7 (4)
C9—C8—N1—Hg1	9.9 (5)	C32—C27—N5—Hg2	17.9 (6)
C8—N1—N2—C1	176.6 (4)	C27—N5—N6—C20	179.7 (4)
Hg1—N1—N2—C1	−9.7 (5)	Hg2—N5—N6—C20	−13.3 (5)
N3—C1—N2—N1	174.9 (4)	N7—C20—N6—N5	175.2 (4)
S1—C1—N2—N1	−7.4 (6)	S2—C20—N6—N5	−7.4 (6)
N2—C1—N3—N4	−178.8 (4)	N6—C20—N7—N8	−178.1 (4)
S1—C1—N3—N4	3.5 (5)	S2—C20—N7—N8	4.4 (6)
C1—N3—N4—C2	−178.5 (4)	C20—N7—N8—C21	177.7 (4)
C3—C2—N4—N3	9.5 (7)	C22—C21—N8—N7	3.1 (7)
C7—C2—N4—N3	−170.6 (4)	C26—C21—N8—N7	−175.8 (4)
N3—C1—S1—Hg1	−162.5 (3)	N7—C20—S2—Hg2	−158.9 (4)
N2—C1—S1—Hg1	20.0 (4)	N6—C20—S2—Hg2	23.9 (4)
C15—C14—Hg1—S1	−42.8 (8)	C34—C33—Hg2—S2	−48.5 (9)
C19—C14—Hg1—S1	140.3 (4)	C38—C33—Hg2—S2	135.3 (5)
C15—C14—Hg1—N1	117.4 (3)	C34—C33—Hg2—N5	115.8 (4)
C19—C14—Hg1—N1	−59.4 (4)	C38—C33—Hg2—N5	−60.4 (4)
C1—S1—Hg1—C14	147.5 (6)	C20—S2—Hg2—C33	147.6 (6)
C1—S1—Hg1—N1	−14.59 (16)	C20—S2—Hg2—N5	−17.98 (17)
N2—N1—Hg1—C14	−158.7 (3)	N6—N5—Hg2—C33	−155.6 (3)
C8—N1—Hg1—C14	14.3 (4)	C27—N5—Hg2—C33	9.5 (4)
N2—N1—Hg1—S1	16.6 (3)	N6—N5—Hg2—S2	21.1 (3)
C8—N1—Hg1—S1	−170.5 (3)	C27—N5—Hg2—S2	−173.9 (4)

### Hydrogen-bond geometry (Å, °)

**Table:**

Cg1 and Cg2 are the centroids of the C2–C7 and C8–C13 rings, respectively.

**Table:**

*D*—H···*A*	*D*—H	H···*A*	*D*···*A*	*D*—H···*A*
C28—H28···Cl1	0.95	2.77	3.598 (5)	146.
C31—H31···F4^i^	0.95	2.53	3.413 (6)	155.
C39—H39A···N2	0.99	2.62	3.558 (6)	158.
C7—H7···Cg1^ii^	0.95	2.54	3.451 (5)	162
C12—H12···Cg2^iii^	0.95	2.70	3.516 (6)	144
C26—H26···Cg2^ii^	0.95	2.69	3.500 (5)	144

Symmetry codes: (i) *x*−1/2, −*y*+3/2, *z*−1/2; (ii) −*x*, *y*, −*z*+1/2; (iii) *x*, *y*−1, *z*.

### Table 2 Short ring-interaction geometries (°, Å)

**Table:**

*Cg*(X)···*Cg*(Y)	*Cg*···*Cg*	Alpha	Beta	Gamma	*Cg*(X)perp	*Cg*(X)perp
*Cg*1···*Cg*4^i^	3.648 (3)	6.3 (2)	21.65	27.98	3.221 (2)	3.391 (2)
*Cg*2···*Cg*3^i^	3.641 (3)	4.1 (2)	27.95	24.04	3.325 (2)	3.216 (2)

For centroids: *Cg*1 = ring C2 – C7, *Cg*2 = ring C8 – C13, *Cg*3 = ring C21 – C26, *Cg*4 = ring C27 – C32; symmetry codes: i = -x,y,1/2-z; Alpha = dihedral angle between *Cg*(X) and *Cg*(Y); *Cg*(X)perp = perpendicular distance of *Cg*(X) on ring Y; *Cg*(X)perp = perpendicular distance of *Cg*(Y) on ring X; Beta = angle *Cg*(X)···*Cg*(Y) vector and normal to ring X; Gamma = angle *Cg*(X)···*Cg*(Y) vector and normal to plane Y;
